# A study of green roof and impact on the temperature of buildings using integrated IoT system

**DOI:** 10.1038/s41598-022-20552-6

**Published:** 2022-09-27

**Authors:** Kuo-Hsiung Tseng, Meng-Yun Chung, Li-Hsien Chen, Lu-An Chou

**Affiliations:** 1 Department of Electrical Engineering, National Taipei University of Technology, Taipei, Taiwan, ROC; 2 Department of Civil Engineering, National Taipei University of Technology, Taipei, Taiwan, ROC; 3 WSP International LLC., Taiwan Branch, New Taipei City, Taiwan, ROC

**Keywords:** Environmental sciences, Engineering

## Abstract

With the rise of environmental consciousness and the evolution of circular cities, the Internet of things (IoT) has been combined with the concept of circular economies to promote the effective control of renewable energy and resources. In this study, a comprehensive IoT system containing front-end device applications, network layer innovations, and cloud platform integrations was used in civil engineering applications. This IoT architecture is presented as a development basis for constructing modular automatic monitoring devices and integrating circular city concepts with the IoT. According to the concept of circular city, green circulation and energy use are systematically integrated and called “green energy”. In addition, the green energy system can be divided into above-ground and underground. The above-ground part uses green roofs and solar panels for research and discussion. The composite solar green roofs of the two are called green roofs, and the comparison of their benefits is discussed on the spot. Narrowband IoT (NB-IoT) technologies were used in this study. The advantages of the developed system were analyzed using measured pH values, air temperatures, soil temperatures, and humidity. The results of this study indicate that constructing a green energy roof can decrease indoor temperatures by 1.5 °C and solar module temperatures by 1.6 °C while increasing power generation; thus, green energy roofs are suitable for tropical regions.

## Introduction

Urban commercial development and dense urban planning have led to a gradual decrease in green coverage, which has resulted in urban heat island effects and wall effects^1^. Increasing the area of urban greeneries and effectively addressing environmental degradation have become urban improvement goals worldwide. This paper suggests that green roofs should be constructed to create urban oases for mitigating warming phenomena. Green roofs can be categorized into potted, thin, and thick green roofs according to the types of plants potted on them and the thickness of the soil on them. The different requirements and environments of the green roofs are assessed to determine the optimal design. The three major green roof structures are described as follows:

1. Potted green roofs: These roofs are ideal for achieving short-term greening goals and have fewer site restrictions^2^. The main feature of potted roofs is localized greening in mostly old buildings. Potted green roofs can be built on roofs with a slope of 10° or less. Furthermore, the carrying capacity of the building must be greater than 250 kg/m^2^^3^. Potted green roofs have higher costs and require more maintenance personnel than do thin green roofs.
2. Thin green roofs: The main function of these roofs is ecological and environmental coordination. Thin green roofs can be built on roofs with soil depths less than 30 cm, low management frequency, and slopes of 45° or less. Shrubs, flowers and grass, and turf can be grown on the aforementioned roofs. Such roofs can effectively decrease the building load capacity. When constructing thin green roofs, the building carrying capacity must be greater than 200 kg/m^2^. These roofs are easy and inexpensive to build; and are suitable for tropical regions, particularly for planting sparse vegetation in only one soil layer^4^.
3. Thick green roofs: These roofs are generally constructed to be used long term, contain recreation spaces, and can support considerable biodiversity, including shrubs and small trees. Thick green roofs can be built on roofs with slopes of 10° or less; however, their construction is complicated and expensive. The required carrying capacity for thick green roofs must be no less than 450 kg/m^2^^5^.

The success or failure of a green roof depends on external and internal factors. The main external factors are climate changes at the site. Two internal factors affect the success of green roofs: (1) the structure of the building and (2) the method and materials used to construct the roof. Therefore, the construction of a green roof requires careful research and planning on five different aspects: the age and carrying capacity of the roof^6^, the waterproofing and water drainage of the roof^7^, rooftop security design, the types of plants potted, and the personnel and costs required for future maintenance. Maintenance is especially vital—regular maintenance and equipment checks must be planned to ensure that green roof functions run normally, allowing for sustainable development and operations. Internet of things (IoT) systems play a major role in the maintenance of green roofs^8,9^. Narrowband IoT (NB-IoT) technologies have a wide range of applications in not only simplifying complexities in middle-tier equipment but also reducing the power consumption of equipment^10,11^. IoT systems can also combine features such as low mobility, low transfer speeds, and high latency and be effectively applied in three major areas: food, housing, and transportation. The effective integration of IoT applications is crucial for the use of IoT technologies to conduct research and create opportunities related to sustainable development and to develop 3E (economy, energy, and environment) models on environment protection, energy management, and economic development. Therefore, this study focused on environmental protection issues and adopted positive green construction practices to create urban green energy roofs and decrease indoor temperatures, outdoor temperatures, and air pollution effectively. This study also monitored environmental green data to achieve the management and maintenance of sustainable development as well as the effective reduction of carbon emissions through the use of green energy. Finally, this study combined green roofs and edible plants together to create a model of economic value.

## Material and methods

### Wireless transmission

Transmission technologies have flourished in the IoT era. These technologies, such as NB-IoT, Long Range (LoRa), SigFox, Zigbee, Bluetooth, WIFI, WiMax, and LTE-MI, can be categorized according to their delays (long or short), transmission distance (long or short), and transmission volumes^12–15^. The transmission technology that fits the requirements of an application is selected according to the environmental category and monitoring content. Low-power wide-area networks (LPWANs) cover a broad range of technologies, including NB-IoT, which is an international communication standard defined by Release 13 of the 3rd Generation Partnership Project (3GPP). NB-IoT is a cellular network architecture that can be directly incorporated into Global System for Mobile, Universal Mobile Telecommunications System (UMTS), or Long Term Evolution (LTE) networks by using operators’ authorized frequency bands and can send messages through existing 4G towers. NB-IoT boasts low power consumption, low transmission volume, and a long transmission distance. Its key features are as follows:

1. The NB-IoT frequency band used worldwide is 700–1800 MHz and that used in Taiwan is 700–900 MHz.
2. NB-IoT has three operation modes: the stand-alone (ST), in-band (IB), and guard-band (GB) modes. The differences between these modes are presented in Table 1^16^. The ST mode is suitable for reframing GSM frequency bands. It exhibits superior performance when using a bandwidth of 200 kHz because the NB-IoT bandwidth is 180 kHz, which allows the use of an additional 10 kHz of the GB. Furthermore, due to the independent power configuration in the ST mode, it does not rely on LTE networks. This mode exhibits high download coverage, speed, and latency as well as low power consumption. The IB mode can be deployed in the LTE band by using a bandwidth of 180 kHz. The high power of LTE networks and the limited download power in the IB mode result in poor download depth coverage. The GB mode is deployed in the unused 180-kHz bandwidth in the GB of LTE services and therefore does not use up existing LTE resources. Due to the high LTE power in the GB mode, this mode is complex and has high tower equipment radiofrequency demands (in theory, the performance of the GB mode is inferior to that of the IB mode)^17^. Thus, when using the IB and GB operation modes, the compatibility of the LTE system with the deployment suggestions must be considered. Priority is first given to the ST and IB modes.
3. The download and upload speeds of NB-IoT are approximately 160–250 and 160–200 kbps, respectively.
4. The latency of NB-IoT is approximately 6–10 s, which is an acceptable range for nonessential or immediate messaging.
5. The transmission distance of NB-IoT is approximately 1–8 km within urban areas and 25 km in rural areas. The maximum transmission distance is approximately 35 km^18^.
6. On standby, an AA battery can power the NB-IoT up to approximately 10 years.
7. According to 3GPP stability standards, NB-IoT can use retransmission data and control signals to enhance the stability of transmission data.
8. Mechanisms based on cellular networks and subscriber identification modules (SIMs) provide messages with favorable security and privacy.
9. Similar to most low-power, long-distance transmission methods, NB-IoT cannot provide mobile transmission.
10. NT-IoT modules are inexpensive. At a cost of approximately US$5 per chip, it enables the rapid maturation of NB-IoT technologies.

**Table 1:**
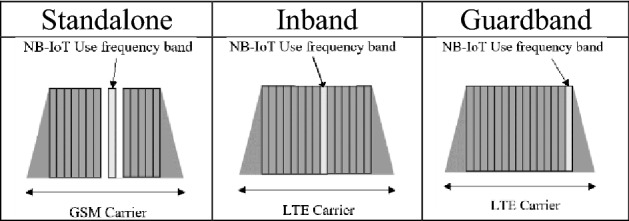
Operation modes of NB-IoT.

The NB-IoT modules used in this study were ENK modules developed by Eagletek. Lierda NB08-01 and NB86-G chips were used in the modules. The older chip, namely Lierda NB08-01, is limited to the frequency band of Chunghwa Telecom and therefore can only scan and connect to Band 8 (900 MHz). This chip is restrictive and cannot be used with other frequency bands or modes. Lierda NB86-G chips are currently used in different devices. Lierda NB86-G is a general-purpose chip and supports different frequency bands and businesses. Lierda NB86-G also supports and can switch between CoAP and MQTT communication protocols at will for different needs; thus, this chip has broad applications. Comparisons between the NB-IoT module chips used in this study is shown in Table 2. Lierda NB08-01 chip has dedicated frequency bands on the spectrum and only scans and connect to specific frequency bands. The module of Lierda NB86-G automatically selects the network and configures the APN for registration after inserting the provider’s SIM card.

**Table 2:** Comparisons between the NB-IoT module chips.

NB-IoT module chips	Lierda NB08-01	Lierda NB86-G
Supported frequency bands	Band 8	Band 1, 3, 5, 8, 20, 28
NB frequency bands used in Taiwan	Band 8 (900 MHz) and 28 (700 MHz)	
Communication protocols	CoAP	CoAP and MQTT
Frequency band of Chunghwa Telecom	Band 8 (900 MHz)	
Operation mode of Chunghwa Telecom	IB mode	

Table 3 presents the NB-IoT transmission prices, transmission frequency band usage, and transmission flow restrictions of two telecom companies in Taiwan. In this study, Chunghwa Telecom was selected as the NB-IoT transmission provider because comparisons indicated that it has more cell towers than and superior platform management to Far EasTone. Furthermore, compared with Far EasTone, Chunghwa Telecom offers larger transmission capacities for the same price in its NB-IoT packages. Thus, the 19.5-MB NB-IoT package of Chunghwa Telecom, which cost NT$25 per month, was selected.

**Table 3:** Comparisons between two NB-IoT providers in Taiwan.

Provider	Chunghwa Telecom		Far EasTone	
3G or 4G	4G		4G	
Number of towers	13,090		10,277	
Transmission frequency band	900 MHz		700 MHz	
Number of towers in Taipei City	1245	1305	1151	1295
Packages	6.5 MB, 19.5 MB		5 MB, 15 MB, 40 MB	
Monthly fees	NT$10, NT$25		NT $10, $25, $60	
Platform management	Yes: Chunghwa IoT Smart Platform, Connectivity Management Platform (CMP)		Yes: CMP	
Monthly platform management fee	Free up to 250 MB per month; NT$10 per month for CMP		Free	

To compare practical communications, in addition to NB-IoT, LoRa communication techniques were used at the same site. LoRa has emerged in recent years and is an LPWAN technology. LoRa equipment is mainly composed of a receiving end and a transmitting end, and enables multipoint communication. The transmitting end consumes more power than the receiving end does; however, in terms of module hardware, no difference exists between the two ends. The advantages of LoRa are that it uses license-free frequency bands, entails a low cost, and can achieve long-distance transmission with low power consumption. The LoRa transmission module adopted in this study contained a Semtech SX1278 chip and had four operating modes: the normal, wake, power-saving, and sleep modes. The sleep mode used the least power. In this mode, the transmission module was unable to receive or send data and must be paired with control board actions. In the power-saving mode, the module could only receive data and must be paired with a LoRa transmission module in the wake mode. LoRa had 32 channels, and its operational frequency bands were in the range of 410–441 MHz. The LoRa transmitting end connected with microcontrollers through the UART protocol. The receiving end transmits data to Raspberry Pi and the server through a USB-to-TTL converter.

Different API functions of the IoT technology of Chunghwa Telecom were used in this study to achieve real-time monitoring and NB-IoT flow management. Furthermore, the exact locations of sensors and complete monitoring details were obtained using the Advantech WebAccess system. These data were then compiled in a MySQL database for facilitating users to perform data analyses. Three systems were used to capture data and transmit them from the Chunghwa IoT Smart Platform to existing systems. Monitoring personnel could determine the current status of devices as well as integrate and compare existing and new systems to overcome the drawbacks of the aforementioned three systems.

### Sensors

The role played by sensors in the IoT is similar to that played by sensory organs in humans. The application of front-end devices has always been a vital aspect of IoT innovation. The integration of different sensors is applied to items that must be tested at relevant sites to achieve the modularization of a front-end system. The devices used in the current study are described in the following text.

**Figure 1:**
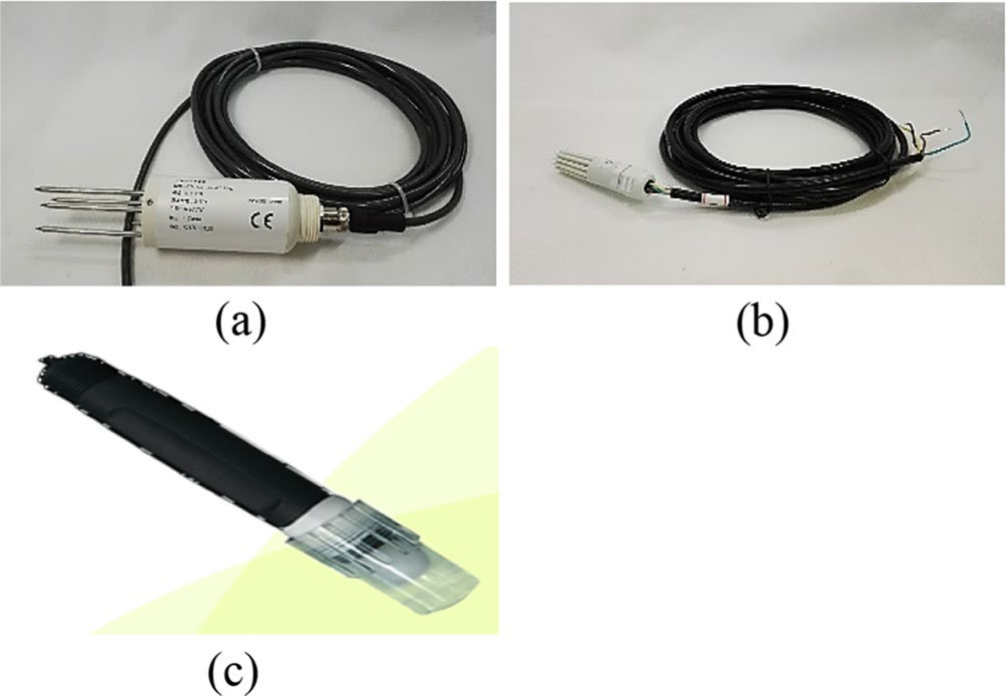
Illustration of the sensors used in this study: (**a**) CSH-11 soil moisture sensor, (**b**) SHT-10 air temperature and humidity sensor, and (**c**) JASP2801 pH sensor.

Soil moisture content is a critical monitoring parameter for green roofs and permeable pavements. Sensors facilitate the achievement of sustainable development in the construction of green roofs, and can also be used in environment monitoring to notify maintenance personnel about when to conduct maintenance tasks and whether the temperature and humidity are appropriate for different types of plants. The CSH-11 soil moisture sensor, which is displayed in Fig. 1a, was used in this study. This sensor had a water and dust resistance level of IP68 and favorable sensor durability. It could be buried in soil for long-term monitoring. The design of this sensor protects it against lightning strikes and cutoff interferences. Furthermore, to prevent the aforementioned sensor from being damaged due to a malfunctioning power terminal, the sensor was designed with current-limiting, overvoltage, and power-reverse-protection measures. This sensor used the RS485 and standard Modbus [Modbus-RTU (remote terminal unit)] protocols and 9600-bps file formats. These specifications and communication protocols can be implemented in most current devices; therefore, the aforementioned sensor can be paired with many module types to completely transmit its data. This sensor has a length of 206 mm, can measure moisture levels between 0 and 100%, and has an error of ± 2%. It can effectively monitor an object under test and measure temperatures of 0–50 °C within a ± 5 °C accuracy. The recommended voltage for the CSH-11 sensor is 12 V_DC_. The needle of this sensor is made from stainless steel, which extends the lifespan of the sensor. The SHT-10 temperature and humidity sensor, which is depicted in Fig. 1b, is designed to measure temperature and humidity changes in air. It uses capacitive technology to measure the atmospheric humidity and an energy gap sensor to measure the temperature. The aforementioned sensor uses industrial COMS technology to provide high-end stability and reliability. Furthermore, this sensor is covered with a plastic shell, which increases the sensor’s lifespan and water repellency. The SHT-10 temperature and humidity sensor uses the I^2^C protocol to transmit signals; therefore, it can be conveniently paired with microcontrollers and is highly compatible with potential future extensions. Furthermore, the SHT-10 sensor can measure temperatures between − 40 and 120 °C and humidity levels between 0 and 100%; thus, it can meet the needs of any application site. Table 4 presents comparisons between the CSH-11 and SHT-10 sensors to highlight the differences between these sensors. In green cities, active campaigns occur for environmental friendliness and considerable quantities of building materials are developed using environmentally friendly materials or recycled waste, which results in the reduction of waste and environmental pollution. However, doubts exist regarding the long-term environmental sustainability of these building materials. Therefore, in this study, a pH sensor was used to monitor a permeable pavement and green rooftop to prevent the permeable pavement from affecting the groundwater quality and corroding the rooftop. The adopted pH sensor (JASP2801) is illustrated in Fig. 1c. A pH sensor made from PPS polymer is suitable for wet soil, and this sensor is calibrated using two-end calibration. The precision, temperature resistance, and pressure resistance of the aforementioned sensor meet the constraints of the research site (Table 5).

**Table 4:** Temperature and moisture sensors.

Model	SHT-10	CSH-11
Function	Air temperature and humidity	Volumetric moisture content in the soil
Signal output	I2C	4–20 mA
Moisture range	N/A	0 ~ 100%
Moisture accuracy	N/A	± 2%
Temperature range	− 40 to 120 ℃	0–50 ℃
Temperature accuracy	± 0.5% ℃	± 0.5 ℃
Humidity range	0–100%	N/A
Humidity accuracy	± 4.5%RH	N/A
Probe material	Plastic	Stainless steel

**Table 5:** Comparisons between different pH sensors.

Model	JASP2801	JASPS2121	JASPA21111
Shell	PPS polymer	PPS polymer	Glass
Aperture size	25*160 mm	25*160 mm	25*160 mm
Measurement range	0–14 pH		
Signal output	± 411.6 mV(4–20 mA)		
Applicable material	Moist soil	Water and wastewater	Seawater and industrial water
Accuracy	± 0.07 pH (pH 5–9), ± 0.1 pH (pH 4–10), ± 0.2 pH (pH 1–3.9, pH 10.1–13) 23 ± 5 °C		
Pressure resistance	0.6 MPa		
Operating temperatures	0–80 °C		

### Construction in a green energy campus

**Figure 2:**
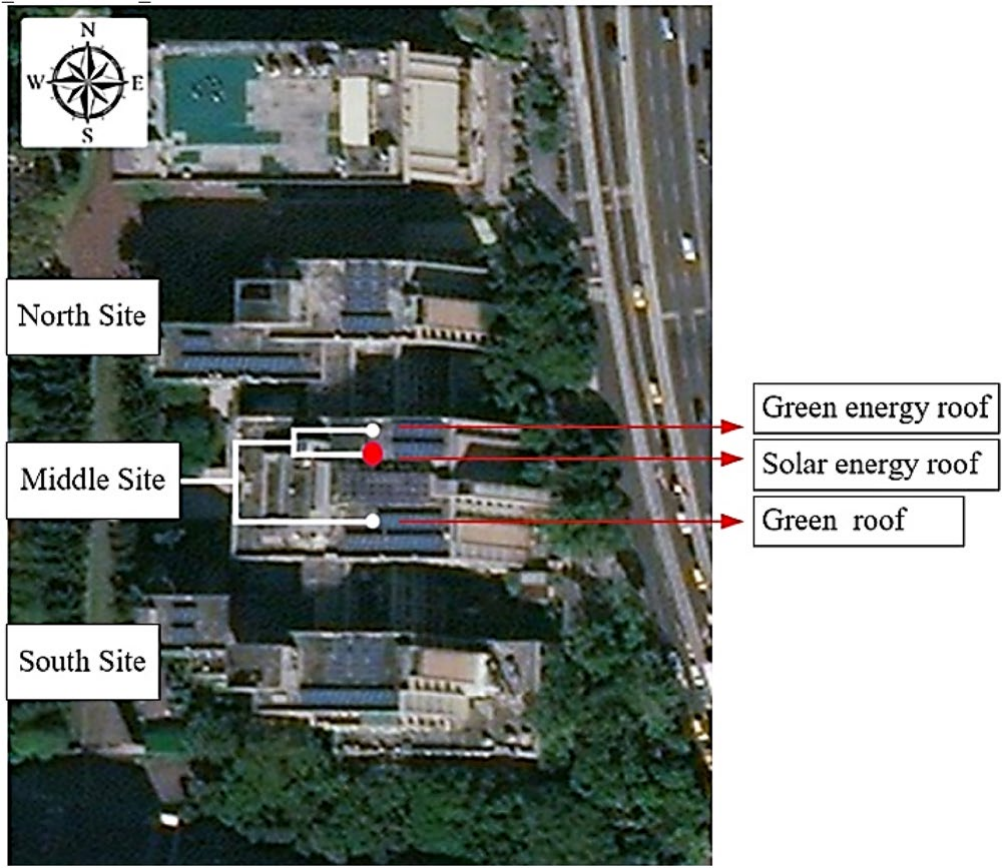
Site selected for the green energy campus. (This satellite imagery was from google map and was modified by adding the words on it).

The site considered for a green roof was the roof of the Integrated Technology Complex at the National Taipei University of Technology as shown in Fig. 2. Solar energy systems and green roofs were integrated to simulate a solar energy roof, green roof, and green energy roof^19^. The differences in indoor and outdoor temperatures and humidity were analyzed. Moreover, pH levels of the soil were monitored to enable the sustainable development and maintenance of the green roof. Finally, the aforementioned three devices were powered using solar energy. The generated solar power was transmitted to the devices and the power mains, which allowed the devices to be self-powered. Also, it has been confirmed that the experimental samples of plants, including the collection of plant material, complied with relevant institutional, national, and international guidelines and legislation with appropriate permissions from Department of Civil Engineering, National Taipei University of Technology, Taiwan, R.O.C. for collection of plant specimens.

#### Simulating a green energy roof and its interior on campus

The systematic planning and construction process involved the analyses and comparisons between different green buildings as well as adjustments to the green roof and angle of the solar cells in accordance with the literature and regulations. Finally, the power usage was matched with the design of the solar energy system to achieve optimal settings. This study was conducted on the roof of the central building of the Integrated Technology Complex at the National Taipei University of Technology, which is represented by a red dot in Fig. 2. The latitude of the site was approximately 25.043°. Square iron tubes were used to secure and support the solar cells in the overall structure^20^. Surfaces were painted with waterproof paint to prevent rusting. The base of the support frame was welded to sheet-iron battens to ensure that the frame was sturdy and secured. For the indoor simulation, the walls were constructed using red bricks. The roof was made from wood laminate and was painted with white water-resistant paint on both sides for insulation. Because Taiwan lies in the subtropical zone and experiences hot summers and rainy winters, white was selected as the paint color because white paint can effectively prevent overheating caused by the sun in the summer^21^. Moreover, the paint was water-resistant to protect the roof against winter rains. The adopted solar panel contained 85-W monocrystalline silicon photovoltaic modules. In Taiwan, solar panels are the most effective at angles between 22° and 25°; therefore, the solar panel used in this study was set at approximately 24°. The red bricks used in this study were made from mixed shale and clay, pressed together, and baked at approximately 900 °C. The aforementioned bricks were strong and durable, and they had robust heat insulation, heat preservation, and sound insulation effects due to their porosity. The aforementioned types of bricks are often used to construct wall structures in buildings. Each brick used in this study weighed approximately 2 kg and had a length of 19.5 cm, a width of 9 cm, and a height of 4.7 cm. The water absorption and pressure resistance of each brick were less than 13% and approximately 300 kgf/cm^2^, respectively. The bricks were laid in a running bond pattern to distribute the load uniformly when simulating the structure of the exterior wall of the building (Table 6).

**Table 6:** Brick categories.

Categories	Pressure resistance	Water absorption	Test
Bricks used in structural walls	300 kgf/cm^2^	Less than 13%	CNS 382
Bricks used in nonstructural walls	200 kgf/cm^2^	Less than 15%	CNS 382

**Figure 3:**
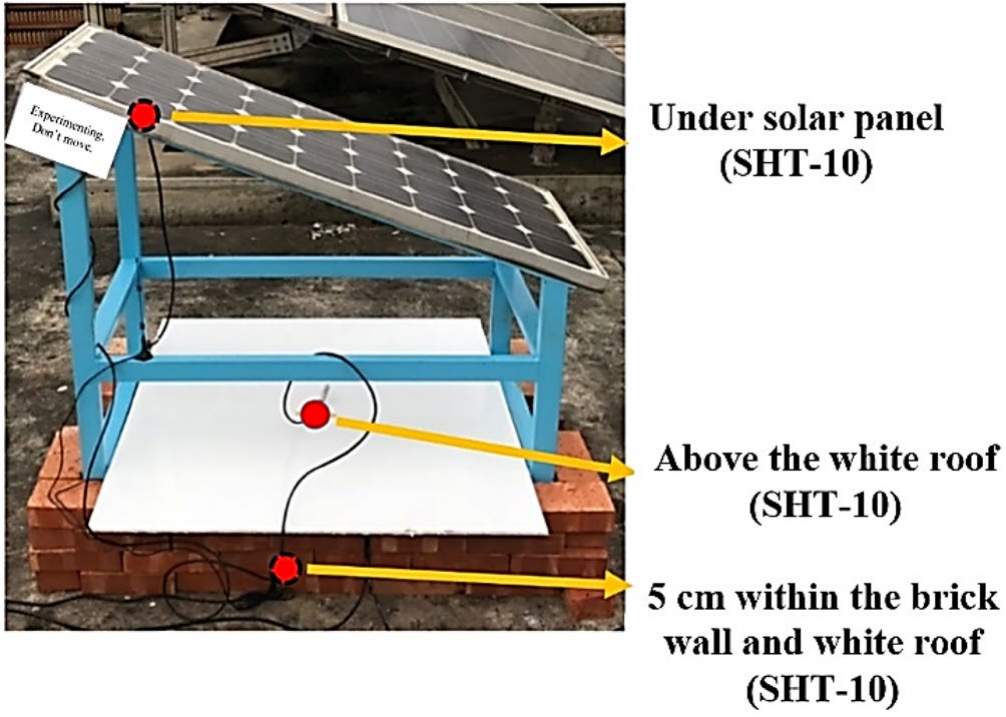
Locations of sensors on the rooftop.

In this study, air temperature and humidity sensors (SHT-10) were selected as the front-end sensors. For the middle-tier transmissions, NB-IoT devices were used to compare the indoor and outdoor simulations. The locations of the sensors are presented in Fig. 3. These locations are described as follows:

- Location 1 is below the solar panel. The sensor at this location is used to sense the current temperature and humidity of the solar panel.
- Location 2 is directly above the white roof. The sensor at this location determines the shading effects of the solar panel on the outdoor temperature.
- Location 3 is 5 cm within the area enclosed by the brick wall and white roof. The senor at this location indicates the changes caused in internal humidity due to the shade from the solar panel.

#### Green rooftop

**Figure 4:**
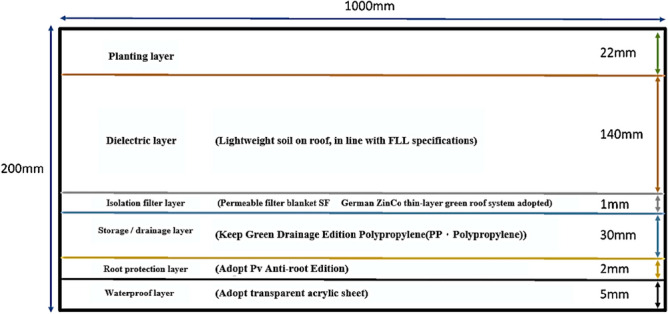
Schematic of a thin green roof.

The experiment setup comprised three sections, namely the thin roof structure at the top, the white roof in the middle, and the red brick interior at the bottom. The features of the thin roof structure are described in the following text and illustrated in Fig. 4.

1. Waterproof layer: The outer frame had a length, width, and height of 1000, 500, and 200 mm, respectively. A 5-mm acrylic board was used as the frame. The waterproof layer was similar to the waterproof layer of a green roof of a house, which prevented water from seeping into the house and causing water leakages. Furthermore, water accumulated inside the layer was discharged through two outlets along the width, which acted similar to rooftop gutter structures.
2. Antiroot layer: This layer was formed from five overlapping pieces of 990-mm black PV root barriers. The overall thickness of this layer was approximately 2 mm. Its function was to prevent plant roots from damaging the roof structure.
3. Water storage/drainage layer: An environmentally green drainage board that could withstand high temperatures and alkalis was constructed from recycled plastic. This drainage board passed the US ASTM pressure test under a pressure of 80 tons/m^2^. This lightweight board alleviated the load on the acrylic board; therefore, the aforementioned drainage board was suitable for being used in thin green roofs. It also minimized the height of the water discharge layer. The dense pores (of approximately 2 cm) in the center of the drainage board exhibited a double-layer honeycomb structure, which had robust drainage. The water storage/drainage layer was affixed with pins along its four sides, which simplified its installation.
4. Insulation and filter layer: This layer comprised two layers of geotextiles to reinforce the filtration effect. Geotextiles are permeable fabrics that can effectively filter soil impurities and reduce soil loss due to drainage. Geotextiles have also been adopted in the thin green roof systems of ZinCo Green Roof Systems (Germany).
5. Stratum medium: The stratum medium contained lightweight soil for plant growth. The organic matter by volume in this layer was less than 20%, and the volume of the inorganic medium was greater than 33%. The specific gravity of the stratum medium was approximately 0.8, which was in compliance with German FLL guidelines. The soil in the aforementioned layer was not easily eroded by wind, and the low specific gravity of the layer reduced the overall weight and load on the roof. The mix of soil particles in the aforementioned layer facilitated the ventilation and drainage of the plant roots, which prevented the roots from becoming waterlogged. The soil coverage in this study was approximately 50 L, which was spread over a height of 150 mm. Comparisons between different soil types is presented in Table 7.
6. Plant layer: This layer was planted with long-day plants, such as potato leaves with shallow roots, because they were convenient and fast to grow and compatible with the concept of edible landscaping on green roofs. Moreover, studies have indicated that potato leaves have low rates of heavy metal residues due to their structure. Potato leaves also have very high economic value. The flavonoids extracted from potato leaves after washing away free radicals through ultrasonic-assisted methods have demonstrated robust inhibitory effects against *Escherichia coli* and *Staphylococcus aureus*.

**Table 7:** Comparisons between different soil types.

Soil properties	General soil	Lightweight soil
Specific gravity	1.5	0.8
Organic matter by volume (%)	Many weed seeds	< 20%
Weight when the soil coverage was spread over a height of 15 cm	225 kg/m^2^	120 kg/m^2^
Specific weight when the soil was saturated with water	2.25	1.2
Medium weight when the soil was when saturated with water	337.5 kg/m^2^	180 kg/m^2^

**Figure 5:**
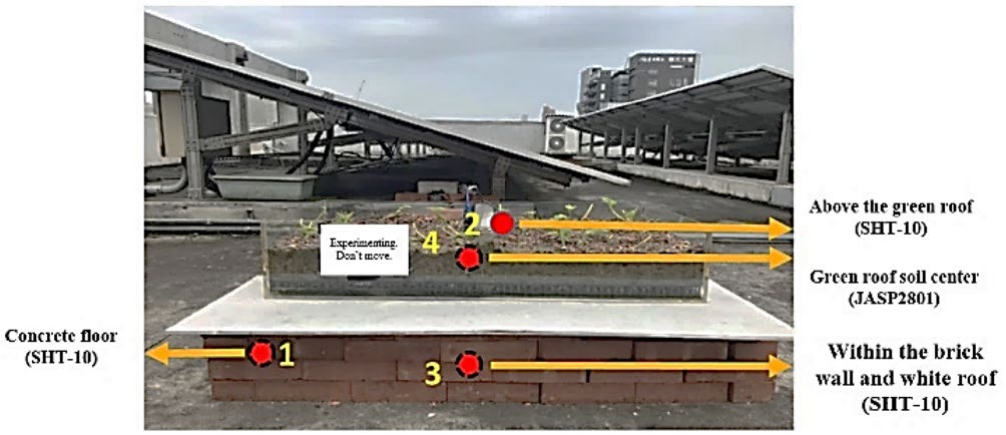
Placement of sensors on the green roof.

In this study, an SHT-10 air temperature and humidity sensor and a JASP2801 pH sensor were selected as the front-end sensors. Moreover, NB-IoT was used as the middle-tier transmission technology. The placement of the devices (Fig. 5) was as follows:

1. Placed above the external concrete floor to detect the temperature and humidity of the original ground surface.
2. Placed directly above the green roof to detect the outdoor temperature and humidity under the shade of the green roof.
3. Placed in the space enclosed by the brick wall and white roof to determine the changes in the indoor temperature and humidity after the green roof was set up.
4. The pH electrode was inserted into the center of the soil to monitor changes in the soil pH and the usage of the sustainable acrylic shell as well as facilitate plant growth.

#### Green energy roof

A green energy roof was designed by combining a green roof with solar modules. The strengths of the green roof and solar modules were compared in indoor and outdoor simulations. Only short-day plants were planted on the green energy roof.

Considering the shade from the solar panel and the design of the thin green energy roof, *Hypoestes phyllostachya* was planted on this roof because *H. phyllostachya* is a short-day plant that prefers warm and humid climates. *H. phyllostachya*, which is also known as the polka dot plant, is an evergreen shrub characterized by spotted leaves. In this study, the “red splash,” “pink splash,” and “white splash” cultivars were planted. *H. phyllostachya* must be trimmed after long periods of cultivation and typically has a height of 20 cm. Air pollution and dust have severe effects in both indoor and outdoor environments. Dust particles larger than 10 µm (PM10) sink under their weight; however, due to the slow speed of descent of these particles, they cause respiratory diseases when inhaled by animals. According to the research of the Department of Horticulture and Landscape Architecture, National Taiwan University, into 50 commonly sold plants, *H. phyllostachya* has a dust retention of 3.02 mg/cm^2^.

**Figure 6:**
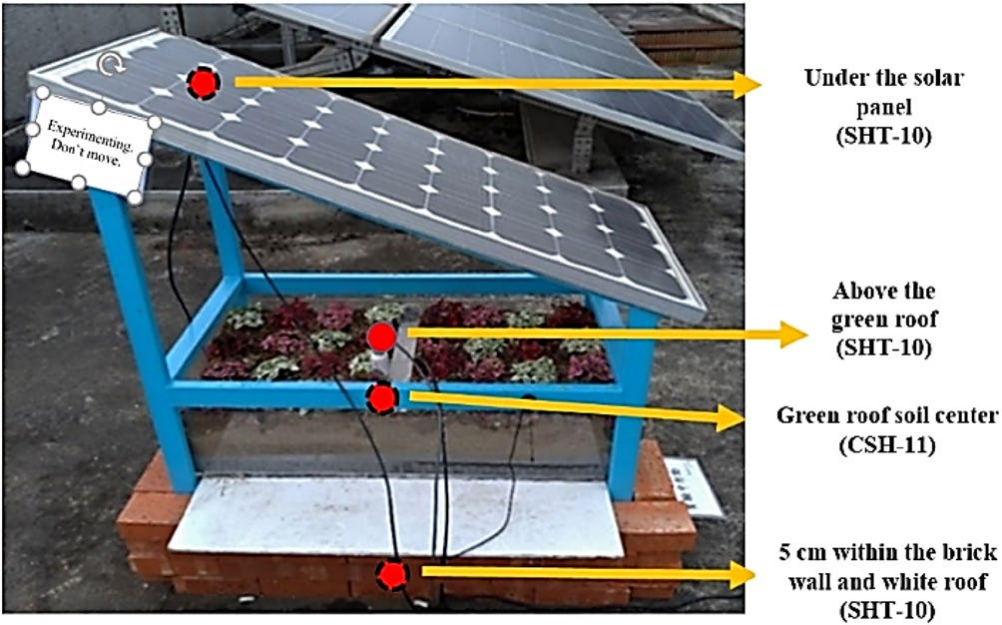
Placement of sensors on the green energy roof.

In the current study, air temperature and humidity sensors (SHT-10) and a soil temperature and moisture sensor (CSH-11) were selected as front-end sensors. For middle-tier transmissions, LoRa devices were selected to compare indoor and outdoor simulations. The positions of the aforementioned sensors are displayed in Fig. 6 and described as follows:

1. Placed directly below the solar panel to sense the temperature and humidity of the solar module.
2. Placed directly above the green roof to simulate the outdoor temperature and humidity under the shade of the green energy roof.
3. Placed in the space enclosed by the brick wall and white roof to sense the indoor temperature and humidity of a green energy roof.
4. A soil temperature and moisture sensor was inserted into the center of the soil to monitor the plants.

**Figure 7:**
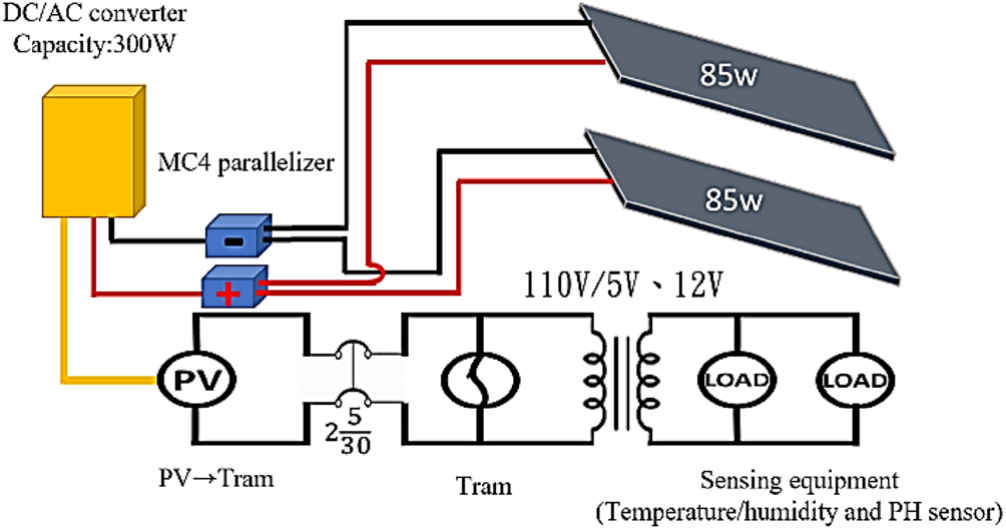
Schematic of the adopted grid-connected solar power system.

On the green energy roof, a solar power system was set up to support the grid-connected use of solar energy. Two 85-W solar panels were connected through an MC4 quick coupler and 4-mm^2^ 1C cables. These cables had an outer diameter of 6 mm and an insulation thickness of 0.5 mm or higher, which guaranteed the protection of outdoor cables. A grid-connected alternating current–direct current transformer pushed the output power into the city-mains end of the device system. The power generated by the solar devices was transmitted to the monitoring system. Figure 7 illustrates a schematic of the power configuration.

## Results and discussion

### Environmental benefit analysis of the mean temperature of the green energy roof

**Figure 8:**
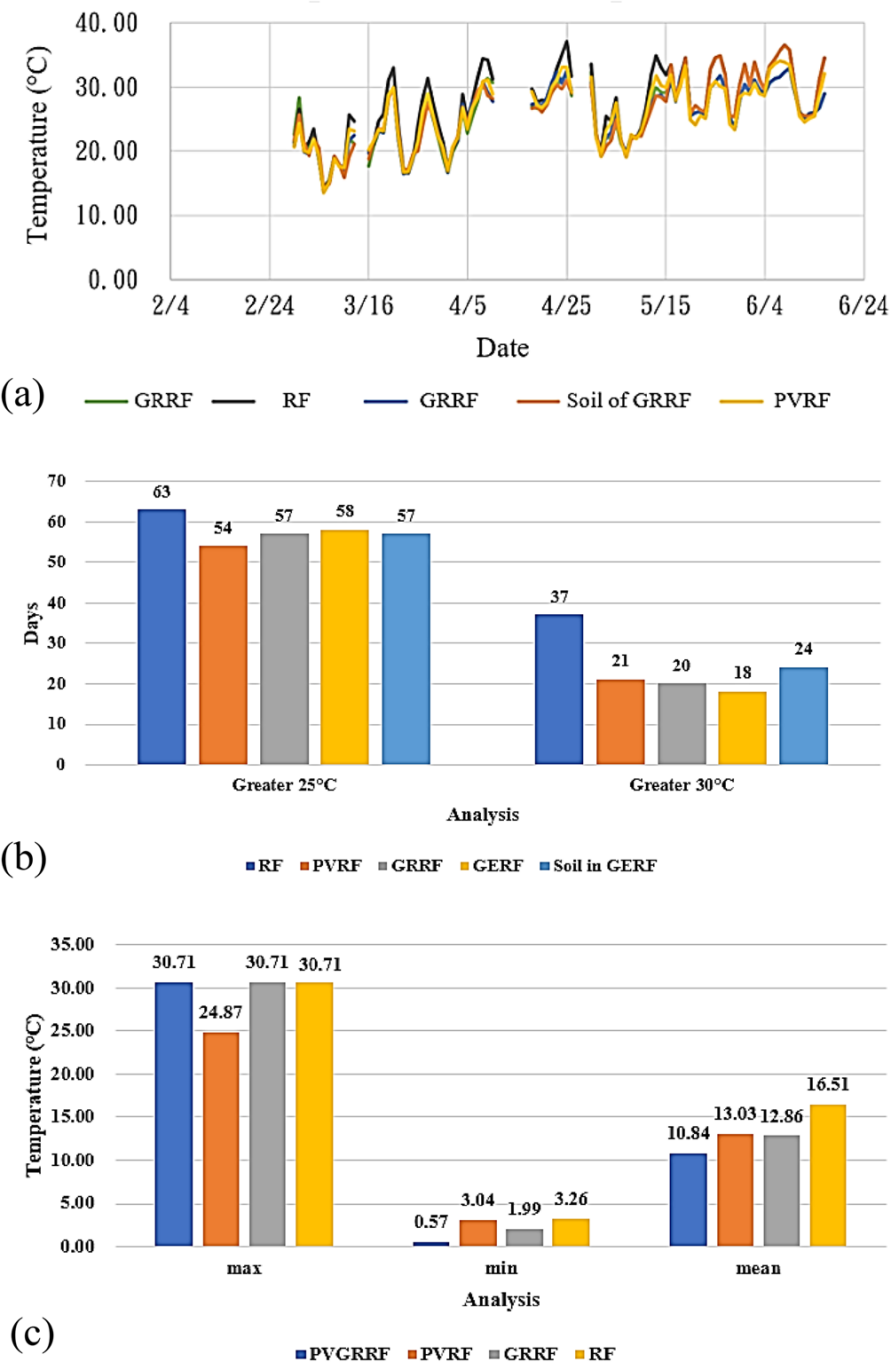
(**a**) Mean daily temperatures, (**b**) number of days with the temperature exceeding 25 °C and 30 °C, and (**c**) temperature changes for each roof type between March 1 and June 16.

An experiment was conducted from March 1 to June 16 (i.e., over 107 days). The mean temperatures of the rooftop surfaces are presented in Fig. 8a. The peak mean temperature of the concrete roof (RF) was 27.18 °C, which was 1.45 °C higher than that of the solar energy roof (PVRF). The temperature difference between the green roof (GRRF) and the green energy roof (GERF) was approximately 1.7 °C (Table 8). The days between March 1 and June 16 with temperatures greater than 25 °C or 30 °C are depicted in Fig. 8b. The concrete rooftop temperatures were greater than 30 °C on 37 days. The installation of a solar energy rooftop and green rooftop significantly reduced this number to 21 days. The mean temperatures of the green energy roof exceeded 30 °C on 18 days, which was lower than the corresponding numbers for the other roofs. The concrete roof exhibited the highest number of days with mean temperatures exceeding 25 °C. By comparison, the green energy rooftop significantly decreased rooftop surface temperatures. The aforementioned results indicate that the construction of solar panels, green roofs, and green energy roofs can effectively decrease the surface temperature on rooftops. These effects are more apparent during higher temperatures. The average daily temperature changes were obtained by calculating the difference between the maximum and minimum temperature changes each day. The results indicated that the concrete roof exhibited the most drastic temperature changes, whereas the green energy rooftop exhibited the smallest temperature changes. The difference between the temperatures of the aforementioned two roofs was approximately 5.7 °C, as depicted in Fig. 8c. Diminishing the temperature changes can delay the aging of sealants, which indirectly extends the lifespan of the rooftop.

**Table 8:** Mean temperatures of the different rooftops.

	RF	PVRF	GERF	GRRF	Soil of GERF
Mean temp (°C)	27.18	25.74	25.50	25.48	25.90
RF (°C)	0	1.44	1.68	1.70	1.28
PVRF (°C)	–	0	0.24	0.26	− 0.16
GERF(°C)	–	–	0	0.02	− 0.40
GRRF (°C)	–	–	–	0	− 0.42

### Peak indoor and outdoor temperatures of the rooftop

**Figure 9:**
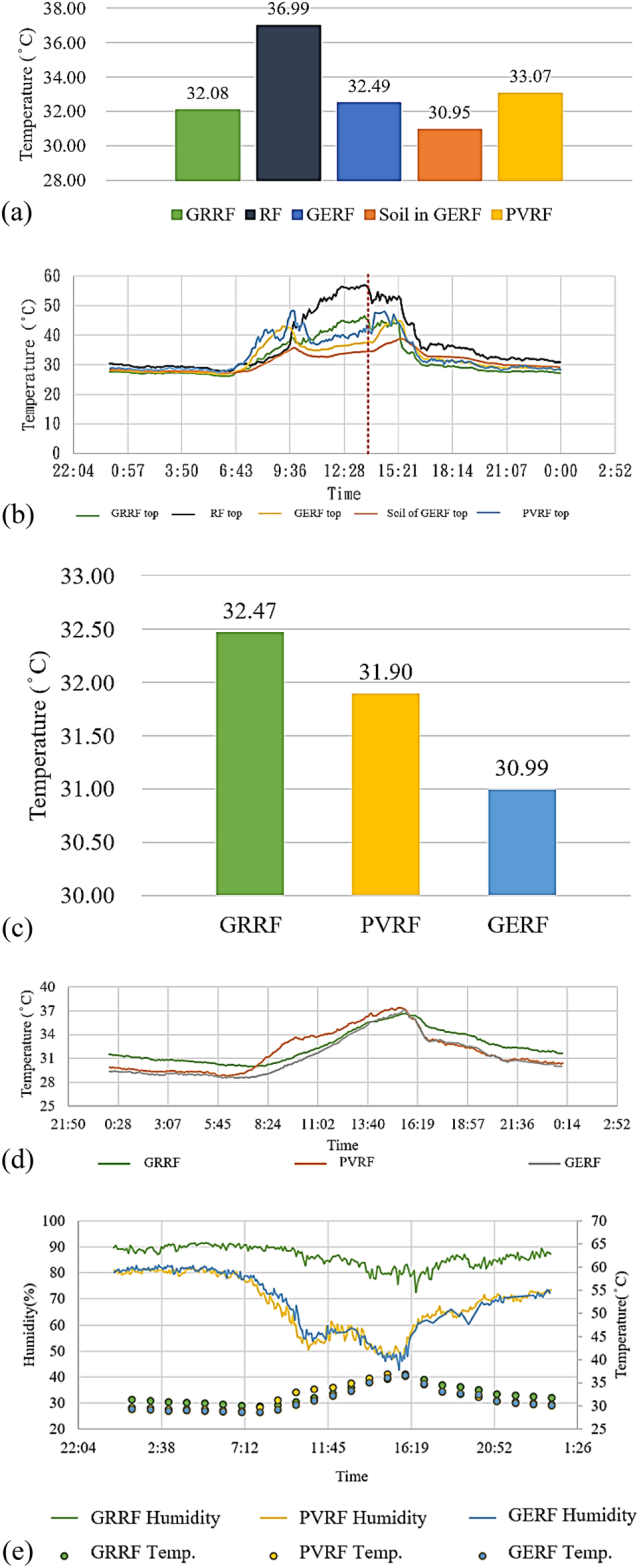
(**a**) Mean surface temperatures, (**b**) surface temperature changes, (**c**) mean indoor temperatures, (**d**) indoor temperature changes, and (**e**) indoor temperature and humidity comparisons for April 25.

Between March 1 and June 16, the mean surface temperature of the concrete roof was the highest on April 25. According to data from the Songshan Station of the Central Weather Bureau (CWB) in Taiwan, on April 25, the mean air temperature was 28.84 °C, no rain occurred, and the highest temperature was 34.5 °C. The mean temperatures of the rooftops are depicted in Fig. 9a. The mean temperature of the surface of the concrete roof was 36.99 °C, which was 4–5 °C higher than those of the surfaces of the other three roofs. This result indicated that the green energy roof significantly and effectively decreased the surface temperature. Furthermore, the soil of the green energy roof had the lowest mean temperature, namely 30.95 °C. Table 9 presents the differences in the mean temperatures of the different roofs on April 25. The highest mean temperature difference (i.e., 4.91 °C) was that between the concrete roof and the green roof. According to the temperature changes for each roof on April 25, the concrete roof had a peak temperature of 59.92 °C at 13:33, which is marked by a red dashed line in Fig. 9a. This temperature was also the highest roof temperature during the period between March 1 and June 16. On April 25, the temperatures on the green roof, green energy roof, and solar energy roof were 46.41 °C, 37.5 °C, and 42.12 °C, respectively. Moreover, on the aforementioned day, the temperature of the soil on the green energy roof was 34.48 °C, with the moisture level being between 7.5 and 11.5%. This result indicated that the soil of the green roof had relatively stable low temperatures, as depicted in Fig. 9b. The peak temperatures of the different roofs are presented in Table 9. Due to its exposure to direct sunlight, the green roof exhibited small differences in its surface temperature. The installation of a solar panel reduced rooftop surface temperatures by 14.8 °C due to the shading effect. The highest temperature reduction was observed for the green energy roof, which was 19.42 °C cooler than the concrete surface. Furthermore, the difference between the temperatures at the soil surface and a 7-cm soil depth was 3.02 °C. The temperature and solar radiation decreased with an increase in the soil depth. The green roof had the highest mean indoor temperature on April 25, which could be attributed to its long exposure to sunlight and heat retention, as indicated by analysis results. The green energy roof benefited from the shade of the solar panel and the heat insulation of the green roof; therefore, the green energy roof was 1.5 °C cooler than the green roof, as depicted in Fig. 9c. This reduction in temperature can decrease the electricity consumption by approximately 0.75°.

**Table 9:** Mean surface and peak temperatures.

Location	Concrete roof	Solar energy roof	Green energy roof	Green roof	Soil of the green energy roof
**Mean surface temperature**					
Mean temperature (°C)	36.99	33.07	32.49	32.08	30.95
Concrete wall (°C)	0	3.92	4.49	4.91	6.03
Solar energy roof (°C)	–	0	0.57	0.99	2.11
Green energy roof (°C)	–	–	0	0.42	1.54
Green roof (°C)	–	–	–	0	1.12
**Peak temperature**					
Peak temperature (°C)	56.92	42.12	37.5	46.41	34.48
Concrete wall (°C)	0	14.8	19.42	10.51	22.44
Green roof (°C)	–	4.29	8.91	0	11.93
Solar energy roof (°C)	–	0	4.62	–	7.64
Green energy roof (°C)	–	–	0	–	3.02

The indoor temperature of the green energy roof was 0.9 °C and 1.48 °C lower than those of the other two roof types, respectively (Table 10). Thus, the green energy roof exhibited favorable effects in decreasing indoor temperatures and energy use. On April 25, compared with the other roofs, the green roof exhibited a gentler curve of overall temperature changes, which indicated its superior temperature maintenance and temperature retention effects during cool periods. On the aforementioned day, the difference between the highest and lowest temperatures of the green rooftop was 6.66 °C. The solar energy rooftop exhibited a difference of 8.64 °C between its highest and lowest temperatures. The green energy roof exhibited smaller temperature changes than did the solar energy rooftop because the green energy roof contained green roof components. Both the green energy and solar energy roofs exhibited rapid temperature decreases. The difference between the highest and lowest temperatures was 8.59 °C, as depicted in Fig. 9d. The largest changes in humidity during the study period were also observed on April 25, which indicated that increases in indoor temperature led to decreases in humidity. Furthermore, the roofs with solar panels exhibited lower humidity and higher indoor comfort than did the green roof, as displayed in Fig. 9e. Table 10 presents the mean humidity of the three roofs. A humidity difference of approximately 17% was observed between the green roof and the other two roofs. This result verifies that green energy roofs combine and multiply the effects of green roofs and solar energy roofs to decrease the temperature and humidity.

**Table 10:** Mean indoor temperature and humidity.

	Green roof	Solar energy roof	Green energy roof
Mean temperature (°C)	32.47	31.90	30.99
Green roof (°C)	0	0.57	1.48
Solar energy roof (°C)	–	0	0.90
Mean humidity (%)	86.33	68.83	69.61
Green roof (%)	0	17.49	16.72
Solar energy roof (%)	–	0	− 0.77

### Comparing indoor and outdoor temperature and humidity (without the mean outdoor humidity chart)

Data were collected on the mean indoor temperatures of the roofs for the period between March 1 and June 16. For the green roof, solar energy roof, and green energy roof, the temperature exceeded 25 °C on 56, 52, and 49 days, respectively, and exceeded 30 °C on 24, 16, and 6 days, respectively. These results indicate that the green energy roof can effectively reduce the number of days with high temperatures (Table 11).

**Table 11:** Mean indoor temperatures and number of days with high temperatures for the different roofs.

	Green roof	Solar energy roof	Green energy roof
Exceeding 20 °C	86 days	84 days	81 days
Exceeding 25 °C	56 days	52 days	49 days
Exceeding 30 °C	24 days	16 days	6 days

**Figure 10:**
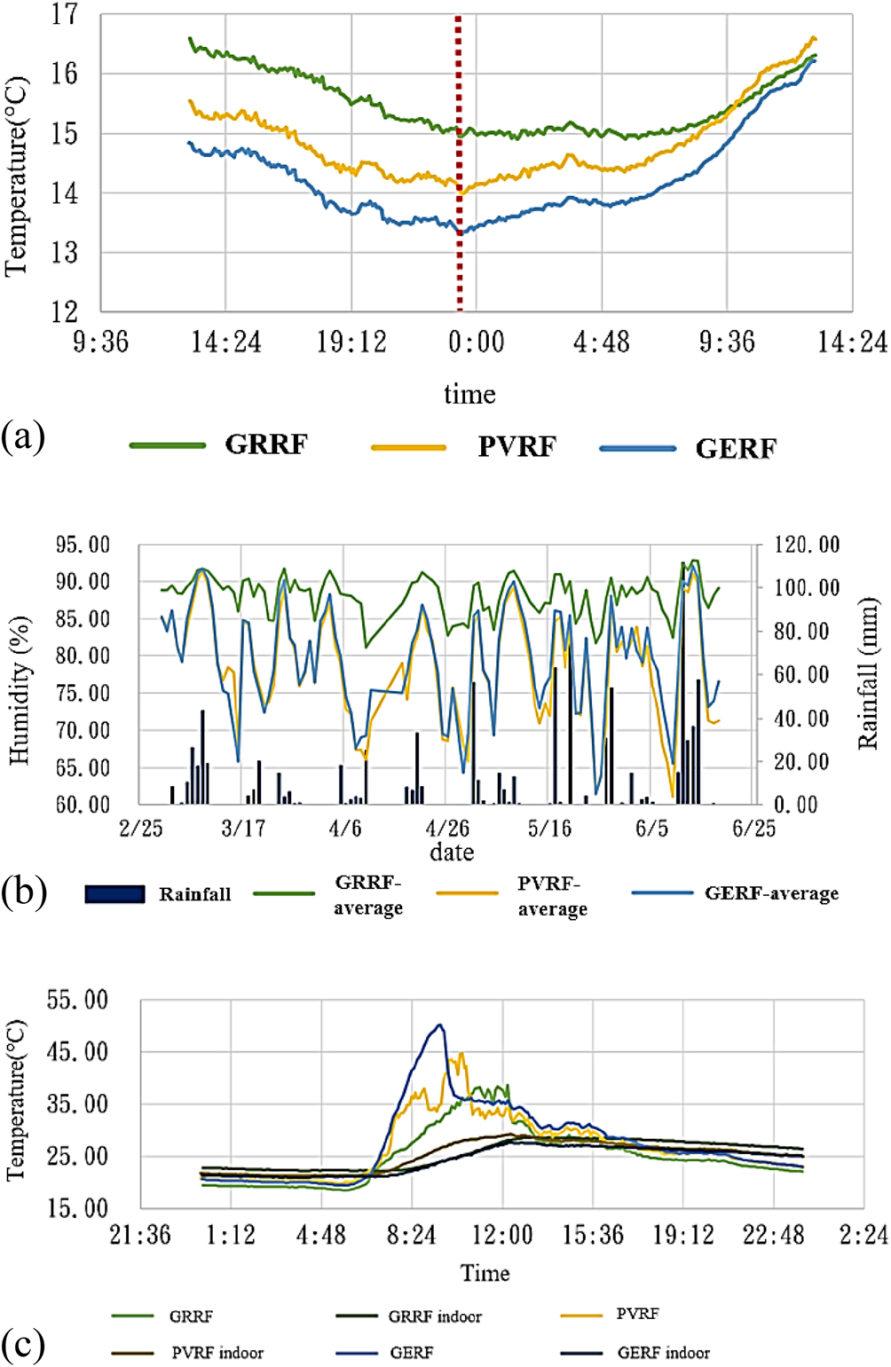
(**a**) Smallest temperature changes, (**b**) indoor roof humidity, and (**c**) indoor–outdoor temperatures on April 6.

During the research period, the lowest temperatures were observed between 13:00 on March 7 and 13:00 on March 8. According to the CWB’s Songshan Station, during the aforementioned time, the mean, highest, and lowest temperatures were 13.41 °C, 15.7 °C, and 12.3 °C, respectively. According to the data, the green roof exhibited the optimal temperature retention and maintained steady temperatures. The green roof exhibited the lowest indoor temperature difference of 1.69 °C. The solar energy and green energy roofs exhibited indoor temperature differences of 2.61 °C and 2.92 °C, respectively, as displayed in Fig. 10a. Furthermore, at the two sites where solar panels were installed, rapid temperature increases occurred possibly due to the generation of heat by the solar energy modules when they were irradiated by sunlight. Comparisons between the mean indoor humidity for the different roofs between March 1 and June 16 are presented in Fig. 10b. The indoor humidity increased in the days immediately following rain as shown in Fig. 10b. The differences in peak humidity among the three roof types were not significant; however, these differences increased with lower humidity. When solar panels were installed, the humidity decreased significantly. The highest indoor–outdoor temperature difference was observed on April 6. The indoor–outdoor temperature differences among the three roof types, as shown in Fig. 10c, indicated large changes in outdoor temperatures and low indoor temperatures. The green roof exhibited the highest indoor temperature of 25.56 °C, followed by the solar energy roof (25.21 °C) and green energy roof (24.4 °C). The green energy roof could maintain low temperatures and therefore is suitable for tropical regions.

### Comparing the temperatures of the solar energy modules

**Figure 11:**
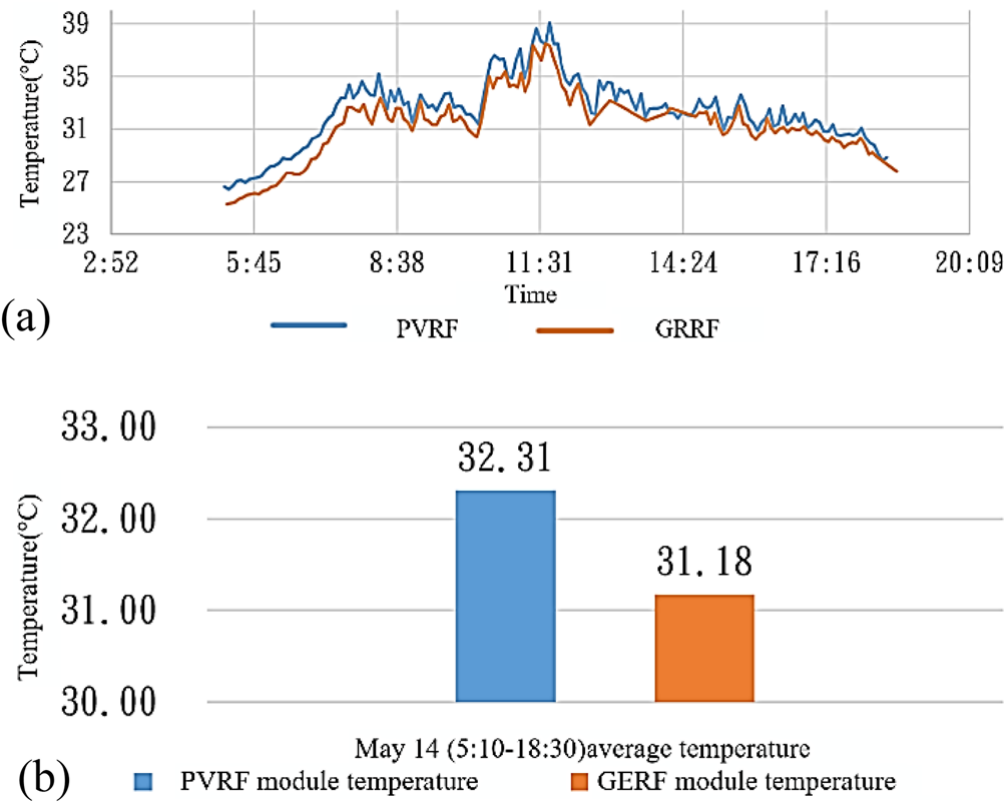
(**a**) Temperatures and (**b**) mean temperatures of the solar panels on May 14.

Figure 11a presents comparisons between the temperatures of the solar panel modules between 05:10 and 06:30 (i.e., between sunrise and sunset as defined by the CWB) on May 14. According to data from the Xinyi Station, the mean air temperature on May 14 was 26.35 °C, with no occurrence of rainfall. The module of the solar energy roof exhibited a peak temperature of 39.09 °C at 11:42. At this time, the temperature of the green energy roof was 37.48 °C, which indicates that the green energy roof effectively decreased temperatures by 1.61 °C compared with the solar energy roof. The mean temperatures during the power generation period were 32.31 °C for the solar energy roof and 31.18 °C for the green energy roof. The difference in the mean temperatures of the aforementioned roofs was 1.13 °C, as depicted in Fig. 11b. A decrease of 1.13 °C in the mean temperature of the solar module can increase the power generation by approximately 0.4%. This result indicates that the green energy roof could effectively lower the temperature of the solar energy module.

Table 12 presents the number of days when the temperatures under the solar panels were greater than 25 °C and 30 °C when using the solar energy roof and green energy roof. Temperatures higher than 25 °C and 30 °C occurred on 2 fewer days when using the green energy roof than when using the solar energy roof. The mean temperature decreased by approximately 0.4 °C when a green roof without plants was adopted.

**Table 12:** Solar module temperatures.

Mean temperatures	Module of the solar energy roof		Module of the green energy roof	
	Exceeding 25 °C	Exceeding 30 °C	Exceeding 25 °C	Exceeding 30 °C
Number of days	62	24	60	22
Mean temperature (22 °C)	26.28		25.87	
Difference	0.4			

### Analysis of the soil pH values

**Figure 12:**
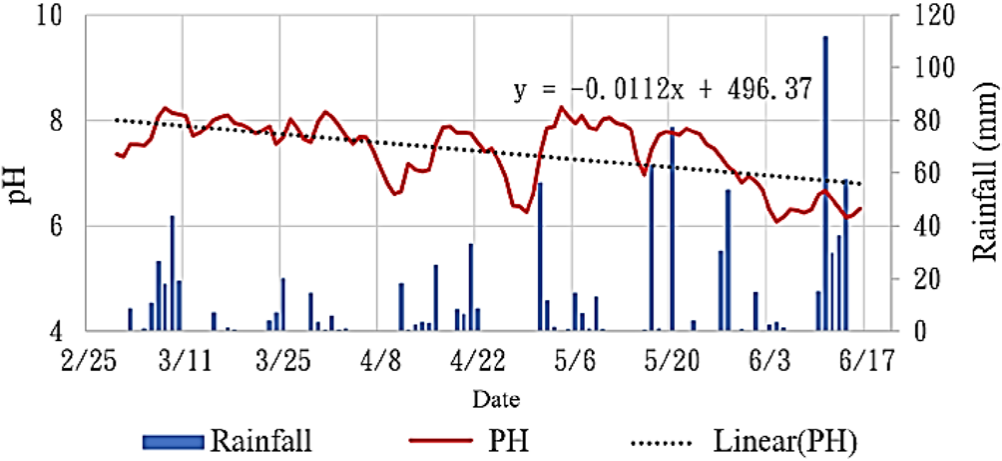
Changes in the pH and rainfall during the research period.

Figure 12 displays the variations in the pH and rainfall during the research period. Loop analysis was conducted to predict the pH trend, with an initial pH of 8.1 as the basis of the prediction. Every 100 days, the pH value dropped by 0.5, and after 550 days, the pH value was approximately 5.6. The warning pH value was set as 5.5. When the pH reaches 5.5, maintenance personnel are notified by a warning to manage the soil pH value and prevent structural damage due to soil hyperacidity and the growth of diseases and insects in the soil environment.

## Discussion

**Figure 13:**
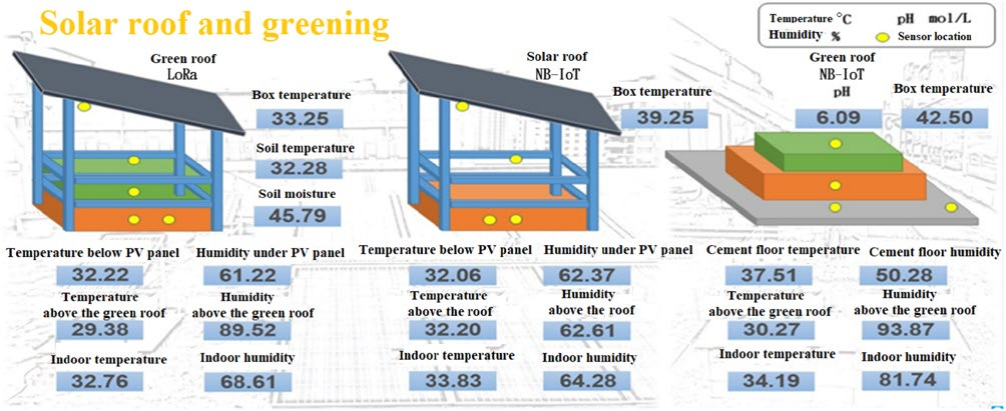
Integrated monitoring management page of the Advantech WebAccess system.

The environment, energy, and the economy are known as the 3E concepts of global development, and these concepts are closely linked. Technological and economic development have resulted in environmental care and sustainable development receiving increasing attention. The United Nation’s Intergovernmental Panel on Climate Change released a special report in 2018 that recommended that global warning should be limited to 1.5 °C or less. This report stated that countries may be threatened by changing environmental trends. In the current study, a comprehensive IoT architecture and the goal and implementation strategy of a circular city were combined with emergent wireless transmission methods to develop devices that integrate greening engineering and automatic monitoring. Laboratory and on-site experiments were conducted through the construction of campus greening projects and small-scale indoor and outdoor simulations. The data collected through the IoT platforms were analyzed to determine the benefits of the constructions. The results indicate that the construction of a green energy roof can decrease indoor temperatures by 1.5 °C and solar module temperatures by 1.6 °C while increasing power generation; thus, these roofs are suitable for tropical regions. In a robust network state, the NB-IoT transmission technology facilitates stable and reliable data transmission. The Advantech WebAccess integrated monitoring portal is illustrated in Fig. 13. This portal displays the temperature and humidity values measured by the three monitoring systems. The aforementioned values are presented with the corresponding locations. Data obtained from each site by using different transmission technologies are displayed on the same page, which allows personnel to understand immediately the state of each item being monitored; thus, the real-time access and analysis of data on intelligent greening projects can be achieved.

## Conclusions

In this study, an integrated IoT platform that incorporated NB-IoT was used to introduce the concept of circular cities to a school campus. Four green energy systems were constructed to evaluate their long-term benefits. These systems were combined with public and private cloud data to measure and verify different parameters. Trend-line judgments were used to predict the maintenance efforts required for these constructions. These predictions enabled the realization of the circular city concept and have considerable environmental and societal value. Different parameters were analyzed and compared for different roof constructions over 107 days between March 1 and June 16. The conclusions of this study are as follows:

1. Effectiveness analyses for green energy roof: The mean surface temperature of the green energy roof was approximately 1.2–1.7 °C lower than those of the solar energy roof, green roof, and concrete roof. The construction of green roofs resulted in the fewest number of days with mean surface temperatures exceeding 30 °C and mean indoors temperatures exceeding 30 °C (6 days). On April 25, namely the day with the highest temperature, the green energy roof maintained the indoor temperature to as low as 30.99 °C, which was 1.0–1.5 °C lower than those under a solar energy roof or green roof; thus, the green energy roof reduced power use by 0.75kWh compared with the other roofs. The green energy roof also decreased the indoor humidity by 17% compared with the other groups. Minimizing indoor–outdoor temperature changes can increase the building lifespan, which indicates the suitability of green energy roofs in tropical regions. Furthermore, compared with the solar module of the solar energy roof, the green energy roof decreased the module temperature by as much as 1.12 °C while increasing the power generation by 0.4%.
2. Effectiveness analyses for the green roof: The mean surface temperatures of the green roof were not considerably different from those of the green energy roof; however, due to direct sun exposure, the green roof exhibited higher mean indoor temperatures than did the other green energy and solar energy roofs. Furthermore, the green roof exhibited the optimal results for the indoor heat and humidity retention in the hottest and coldest climates; therefore, this roof is recommended for regions in high latitudes with cold and dry climates.
3. The pH values of the green roof were monitored, with the warning value set as 5.5. First, rainfall on the green roof led to changes in the pH values. Loop analysis was conducted to predict the soil pH trend. After 550 days, the pH value at the test site fell to 5.6. To prevent acidity from causing structural damage, the dissolution of toxic metals in water, and the growth of viruses, an alert was sent out. CWB data were compared against the experimental data, the coefficient of correlation between these data was determined to be 0.97 by using the CORREL function in Excel. This result indicated that the experimental data were reliable and stable. The protective and predictive management and maintenance of the constructed devices will be continued in the future.
4. Advantages of building green roofs: It can increase plant diversity and provide animal/plant habitats. It can increase the urban water permeability, because the green roof medium layer and storage/drainage layer can effectively retain water, reduce surface runoff, and prevent water flow from entering the drainage system quickly and causing flooding. Temperature control is also available. In summer, the floor temperature can be reduced by up to 2 °C in the room, while in winter, the thermal insulation function keeps the temperature indoors. In addition, the utilization rate of cooling and heating equipment is reduced, which can save 10% of energy.

## Data Availability

The data used to support the findings of this study are included within the article.
